# Bioinformatic Analysis of the Proteome in Exosomes Derived From Plasma: Exosomes Involved in Cholesterol Metabolism Process of Patients With Spinal Cord Injury in the Acute Phase

**DOI:** 10.3389/fninf.2021.662967

**Published:** 2021-07-09

**Authors:** Chunshuai Wu, Jinjuan Yu, Guanhua Xu, Hong Gao, Yue Sun, Jiayi Huang, Li Sun, Xu Zhang, Zhiming Cui

**Affiliations:** ^1^Department of Spine Surgery, Nantong First People's Hospital, The Affiliated Hospital 2 of Nantong University, Nantong, China; ^2^Department of Administrative Office, The Third People's Hospital of Nantong, Nantong, China

**Keywords:** spinal cord injury, bioinformatic analysis, exosome, cholesterol metabolism, proteomics

## Abstract

Spinal cord injury (SCI) is a common but severe disease caused by traffic accidents. Coronary atherosclerotic heart disease (CHD) caused by dyslipidemia is known as the leading cause of death in patients with SCI. However, the quantitative analysis showed that the cholesterol and lipoprotein concentrations in peripheral blood (PB) did not change significantly within 48 h after SCI. Due to the presence of the Blood spinal cord barrier (BSCB), there are only few studies concerning the plasma cholesterol metabolism in the acute phase of SCI. Exosomes have a smaller particle size, which enables them relatively less limitation of BSCB. This study uses exosomes derived from the plasma of 43 patients in the acute phase of SCI and 71 patients in the control group as samples. MS proteomics and bioinformatics analysis found 590 quantifiable proteins, in which 75 proteins were upregulated and 153 proteins were downregulated, and the top 10 differentially expressed proteins are those including downregulating proteins: HIST1H4A, HIST2H3A, HIST2H2BE, HCLS1, S100A9, HIST1H2BM, S100A8, CALM3, YWHAH, and SFN, and upregulating proteins: SERPIND1, C1QB, SPTLC3, IGHV4-28, C4A, IGHV4-38-2, IGHV4-30-2, SLC15A1, C4B, and ACTG2. Enrichment analysis showed that the largest part of proteins was related to cholesterol metabolism among the downregulated proteins. The main components of cholesterol [ApoB-48 and ApoB-100 increased, ApoA-I, ApoA-II, ApoA-IV, ApoC, ApoE, and Apo(a) decreased] were changed in exosomes derived from plasma of patients. ELISA analysis showed that some components were disordered in the acute phase of SCI. These results suggested that the exosomes might be involved in cholesterol metabolism regulation in the acute phase of SCI.

## Introduction

Spinal cord injury (SCI) is a common and severe disease, and the morbidity is continuously increasing, especially along with the increasing amount of traffic accidents, which usually cause physical, psychological, and social impairment (Byra, 2016; Nagoshi et al., 2016; Casper et al., 2018). Traumatic SCI is always divided into primary and secondary injuries according to their different mechanisms and time related to neurological injury. Direct trauma (primary injury) mainly to the vertebrae, such as a cord contusion or compression, initiates a secondary injury cascade of pathophysiological events, including blood flow interruptions, alterations of electrolytes, neurotransmitter accumulations, the release of oxygen free radicals, protein/lipid oxidation, DNA damage, inflammations, and edema formations (Ahuja et al., 2016; Lee et al., 2018). The focus of primary injury is accident prevention, and then the only available therapeutic target for traumatic SCI is to minimize the secondary injury (Lee et al., 2018), mainly including methylprednisolone and the early surgical decompression of the spinal cord (Badhiwala et al., 2018).

The secondary injury of traumatic SCI is temporally divided into four phases, namely acute phase (within 48 h), subacute phase (2–14 days), intermediate phase (14 days−6 months), and chronic phase (more than 6 months) (Badhiwala et al., 2018; Wang et al., 2019). In the acute phase of SCI, due to the existence of the blood spinal cord barrier (BSCB), the microenvironment changes are almost around the lesion site, including hemorrhage, ischemia, tissue edema, metabolic and mitochondrial dysfunction, loss of ionic homeostasis, excitotoxicity, cell death by apoptosis or necrosis, and oxidative stress and excessive generation of free radicals and fatty acids (Kim et al., 2017; Rodrigues et al., 2018; Wang et al., 2019). After 48 h (which lasts until 2 weeks), these pathological changes disrupt the integrity of BSCB and increase its permeability (Pineau and Lacroix, 2007).

Due to the presence of the blood–brain barrier (BBB) and the BSCB, there has been very limited research on peripheral blood (PB) related to SCI. Previous serological analysis showed that total cholesterol (TC), low-density lipoprotein cholesterol (LDL-c), very low-density lipoprotein (VLDL-p), and triglyceride (TG) were increased in patients with SCI, while high-density lipoprotein cholesterol (HDL-c) was reduced (Koyuncu et al., 2017; La Fountaine et al., 2017). Abnormal changes in lipid metabolism are thought to be related to the long-term bed rest of patients. Cerebrospinal fluid (CSF) is not routinely collected in the clinical management of patients with SCI, human biomarker studies examining CSF have been relatively limited and typically involve a relatively small number of patients. A literature review about biomarkers for SCI, based on seven CSFs of patients with SCI, was evaluated to measure UCH-L1, SBDP, MBP, and GFAP (Yokobori et al., 2015). ELISAs and Luminex bead assays of CSF were used to characterize the expression level of a series of inflammatory cytokines (IL-6, IL-8, and MCP-1) and structural proteins (tau, S100β, and GFAP) (Kwon et al., 2010). Although, it is a challenge to detect effective protein-related information in serum or plasma samples due to the significant sample depletion and low expression of SCI-associated biomarkers. Some research investigated certain signals within PB of the patients with SCI, such as GFAP, pF-H, neuron-specific enolase (NSE), inflammatory cytokines, macrophage migration inhibitory factor (MIF), and high mobility group box 1 (HMGB1) (Ahadi et al., 2015; Bank et al., 2015; Kuhle et al., 2015; Papatheodorou et al., 2017).

Given the above limitations, this study attempted to use exosomes in PB as samples due to their smaller particle size, easier isolation, and special characteristic (Lotvall et al., 2014). Exosomes play important roles at physiological and pathophysiological central nervous system (CNS) barriers. Exosomes serve as biomarkers of SCI, and they are considered to have a large potential for treatment because of their relatively less limitation of BSCB in 48 h (Matsumoto et al., 2017; Zhao and Zlokovic, 2017; Gao et al., 2018; Ramirez et al., 2018). In addition, the advanced proteomics and bioinformatics analysis techniques allow us to explore the mysteries in exosomes after SCI. At present, exosomes related studies in SCI show that exosomes function as anti-inflammatory, protecting BSCB, improving microcirculation, promoting angiogenesis, regulating neuronal apoptosis and differentiation, and promoting neurological function (Huang et al., 2017; Sun et al., 2018; Lu et al., 2019; Xu et al., 2019). However, most of the current research is focused on animal experiments. The expression of the protein in exosomes is slightly low, which only represents part of the pathological change process after SCI. But these little change in plasma exosomes is likely to be an important feature of pathological changes in the acute phase after SCI.

This study uses plasma from patients with SCI in the acute phase as samples, attempting to reveal the mystery of exosomes derived from the plasma of patients with SCI *via* proteomics and bioinformatics analysis. This study explored whether the cholesterol and lipoprotein changed during the acute phase of SCI. These results indicate that the abnormal lipid metabolism process after SCI is not entirely caused by long-term bed rest but partly SCI itself. The exosomes derived from plasma were involved in the lipoprotein metabolism process of patients with SCI in the acute phase but did not cause the changes in plasma lipoprotein and cholesterol concentrations.

## Materials and Methods

### Sample Collection and Data Analyses

This study meets the requirements of the Chinese “Code of Ethical Review of Human Biomedical Research” (DB31/T899) and was given official approval by the Ethics Committee of the Nantong First People's Hospital, and all the patients were informed and signed a paper informed consent form for scientific research. The experimental group in this study was patients with both vertebral fractures and SCI because patients with only SCI are relatively rare and much more common are patients with both vertebral fractures and SCI caused by violence in the clinic. To exclude the constitutive influence on the expression of exosomes, we choose patients with only vertebral fractures as the control group, not the healthy ones. The control group has no other comorbidities, including neurological dysfunction, numbness, and dyskinesia of lower limbs, difficulty urinating, or sexual dysfunction. According to the imaging data of the patient (MRI, CT, and X-ray), clinical symptoms (Limb movement and skin sensation), and professional scoring standards (SCI: ISNCSCI and ASIA; Vertebral fracture: TLICS), two spine surgeons and two radiologists will divide the patients into experimental or control groups. Experimental groups (Marked as Sbef48h):43 persons in total, 13 females and 30 males, average age 58 ± 13.0 years. Control groups (Marked as Cbef48h): 71 persons in total, 24 females and 47 males, average age 45 ± 10.5 years.

All enrolled patients underwent surgery and had no history of long-term use of any drugs before the injury. All patients were not diagnosed with any diseases by professional doctors, including dyslipidemia, osteoporosis, nervous system diseases, tumors, and cardiovascular diseases. In both the experimental group and the control group, blood samples were taken immediately after hospitalization. The time limitation for collecting the blood samples was within 48 h after the injury, without any treatment or medication. One part of the blood samples (5 ml) was sent to the laboratory within 30 min to detect the plasma lipid composition, and the other part (20 ml) was centrifuged (9,000 rpm, 4°C, 5 min) for the supernatant (plasma), and then was stored in a deep freezer at −80°C.

### Purification and Identification of Exosomes

Exosomes were extracted from pooled plasma samples through the QIAGEN exoEasy Maxi kit (76064), strictly following the instructions of the manufacturer. The integrity of exosome vesicle morphology by using electron microscopy was observed. The PBS buffer that dissolves the exosomes were prefiltered with a 0.2 um aqueous filter to remove impurities such as crystals. About 20 μl of the PBS to resuspend the precipitated sample was taken and dropped on the copper mesh, and then it was allowed to stand for 30 min. The exosome solution with filter paper was aspirated. Staining with 2% phosphotungstic acid (PH5.0) for 60 s, and then the samples were examined by transmission electron microscopy (TEM). Nanoparticle tracking analysis technology was used to detect the size of exosomes (Model: PARTICLEMETRIX ZETAVIEW, Temperature: 20.71°C sensed, pH 7.0, entered Conductivity: 15,000.00 μS/cm sensed). Western blot was used to detect the surface proteins of exosomes (antibody: CD9 and CD63, 1:1,000, Abcam). Each experiment was repeated three times.

### Quantitative Proteomics Analysis of Exosome Proteins

About 1% protease inhibitor was added to the sample and ultrasonic lysis was used to extract protein. Trypsin was added at 1:50 trypsin-to-protein mass ratio for the first digestion overnight and 1:100 trypsin-to-protein mass ratio for a second 4 h-digestion. The peptide was desalted by Strata X C18 SPE column (Phenomenex) and vacuum dried. The peptide was reconstituted in 0.5 M TEAB and processed according to the protocol of the manufacturer for the TMT kit/iTRAQ kit. The peptides were subjected to NSI source followed by tandem mass spectrometry (MS/MS) in Q ExactiveTM Plus (Thermo) coupled online to the UPLC. The m/z scan range was 350–1,800 for a full scan, and intact peptides were detected in the Orbitrap at a resolution of 70,000. The resulting MS/MS data were processed using the Maxquant search engine (v.1.5.2.8). Quantitative proteomics analysis was repeated three times.

### Bioinformatics Analysis

We annotate proteins through Gene Ontology from the UniProt-GOA database (http://www.ebi.ac.uk/GOA/). The ontology covers three domains: cellular component, molecular function, and biological process. We use the KEGG pathway database to annotate protein pathways: first, use the KEGG online service tool KAAS to annotate the submitted proteins, and then use the KEGG mapper to match the annotated proteins into the corresponding pathways in the database. We use the software WoLF PSORT, which predicts subcellular localization, to annotate the subcellular localization of the submitted proteins. We first collected the functional classification information and corresponding *P*-values enriched by the protein groups were used, and then screened out the functional categories that were significantly enriched (*P* < 0.05) in at least one protein group. The *P*-value data matrix obtained by screening is first subjected to logarithmic transformation with –log10, and then the transformed data matrix is applied to each function classification by Z transformation. Finally, the data set obtained after the Z transformation is used for the hierarchical clustering (Euclidean distance, average connection clustering) method for unilateral cluster analysis. The clustering relationship is visualized using the heat map drawn by the function heatmap.2 in the R language package (Gplots).

### ELISA and Statistical Analysis

The ELISA kit was purchased from CUSABIO (ApoA-I, ApoA-II, ApoA-IV, ApoC, ApoE, Apo(a), ApoB-100) and Elabscience (ApoB-48). The experimental samples were the plasma of patients (Sbef48h and Cbef48h were 40 samples in each group). The ELISA experiment was carried out following the instructions of the manufacturer, and the dilution concentration was as follows(ApoB-100, 1:1,000; ApoB-48, 1:500; ApoA-I, 1:4,000; ApoA-II, 1:2,000; ApoA-IV, 1:1,000; ApoC, 1:20). The expression of Apo(a) is related only to LP(a), so we did not check its concentration in plasma. The detection wavelength was 450 nm. The data of OD values and cholesterol concentrations were analyzed using the Independent-Sample Test through SPSS20. The software used to make the chart is GraphPad Prism 6.01 and Adobe Photoshop CS6. *P* < 0.05 was considered significant.

## Results

### Cholesterol and Lipoprotein Concentrations Were Not Changed Significantly in the Acute Phase of SCI

In this study, we performed a quantitative analysis of the cholesterol and lipoprotein in PB of patients with SCI in the acute phase. Within 48 h after SCI, the cholesterol and lipoprotein concentrations did not change significantly, including the clinically common indicators TG, TC, HDL-c, LDL-c, ApoA, ApoB, and Lp(a) (Table 1). This result showed that cholesterol and lipoproteins were at normal levels, which is different from the previous research results. Is it because BSCB has not lost its barrier function in the acute phase of SCI, or is it because SCI does not cause changes in cholesterol and lipoproteins in PB? This study chose exosomes as the research vehicle to explore answers to these questions.

**Table 1:** The expression of cholesterol and lipoprotein concentrations.

**Group**		**Mean**	**SD**	***P*-value**
TG	Sbef48h	1.673	1.796	0.248
	Cbef48h	1.385	0.833	
TC	Sbef48h	4.013	0.904	0.707
	Cbef48h	4.081	0.986	
HDL-c	Sbef48h	1.211	0.411	0.600
	Cbef48h	1.254	0.438	
LDL-c	Sbef48h	2.263	0.737	0.144
	Cbef48h	2.487	0.864	
ApoA	Sbef48h	1.140	0.515	0.087
	Cbef48h	1.026	0.168	
ApoB	Sbef48h	1.000	0.724	0.787
	Cbef48h	0.966	0.479	
Lp(a)	Sbef48h	1.372	1.691	0.257
	Cbef48h	1.775	2.030	

### Characterization of Human Plasma Delivered Exosomes After SCI

**Figure 1:**
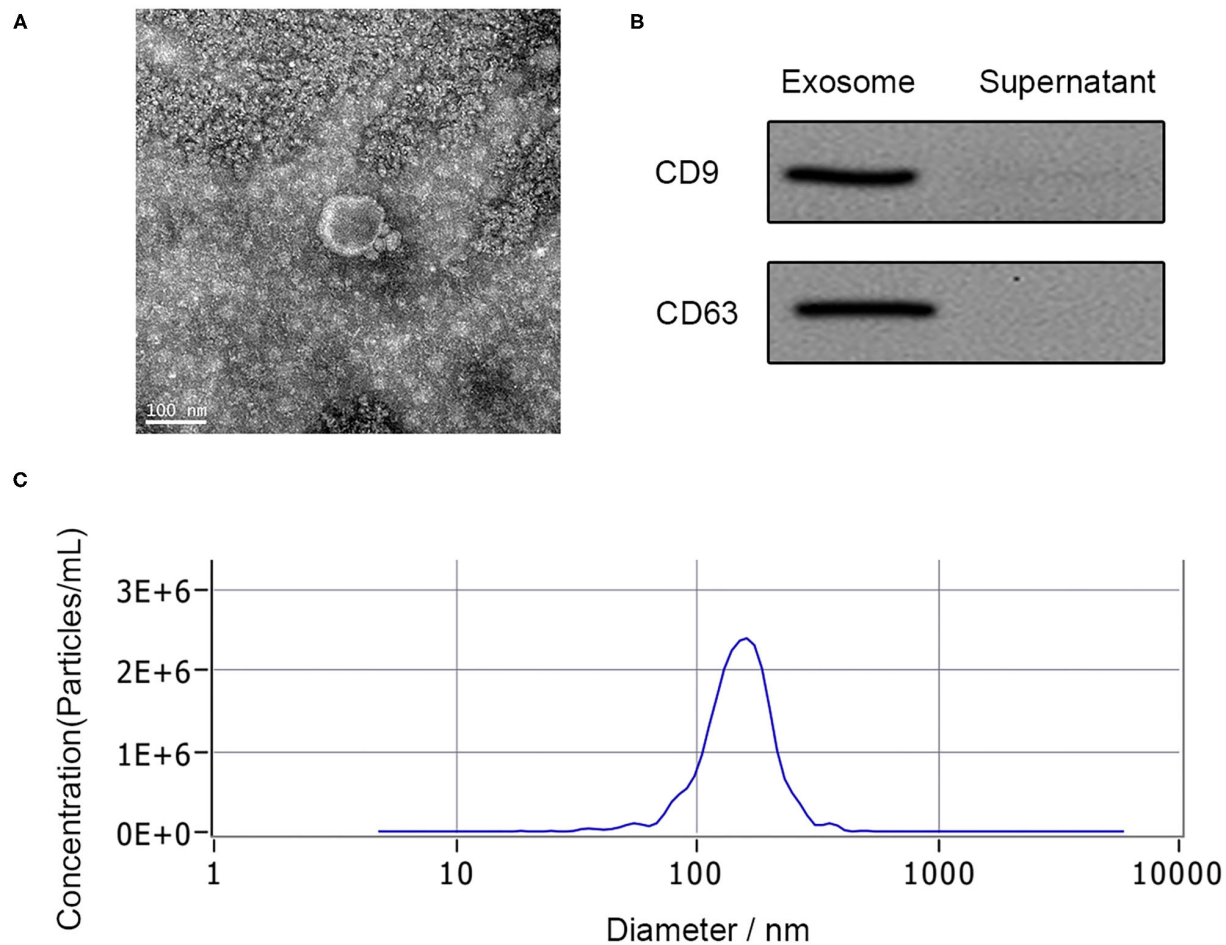
Characterization of human plasma delivered exosomes. **(A)** The morphology of exosomes by TEM. **(B)** The specific exosomes surface markers (CD9 and CD63) by western blotting. **(C)** Nanoparticle tracking analysis of exosomes.

Exosomes have relatively less limitation of BSCB within 48 h after SCI, which makes them easier to be secreted to PB. Exosomes derived from the PB plasma of the patient were analyzed by TEM, nanoparticle tracking analysis, and western blotting. TEM observation showed the spherical vesicles and a typical cup shape in plasma exosomes (Figure 1A). The specific exosome surface markers including CD9 and CD63 were positive in plasma exosomes according to western blotting results (Figure 1B). Nanoparticle tracking analysis revealed that the diameter size distribution of these particles varied from 30 to 150 nm in all samples (Figure 1C).

### Overview of Differentially Expressed Proteins in Human Plasma Delivered Exosomes

**Figure 2:**
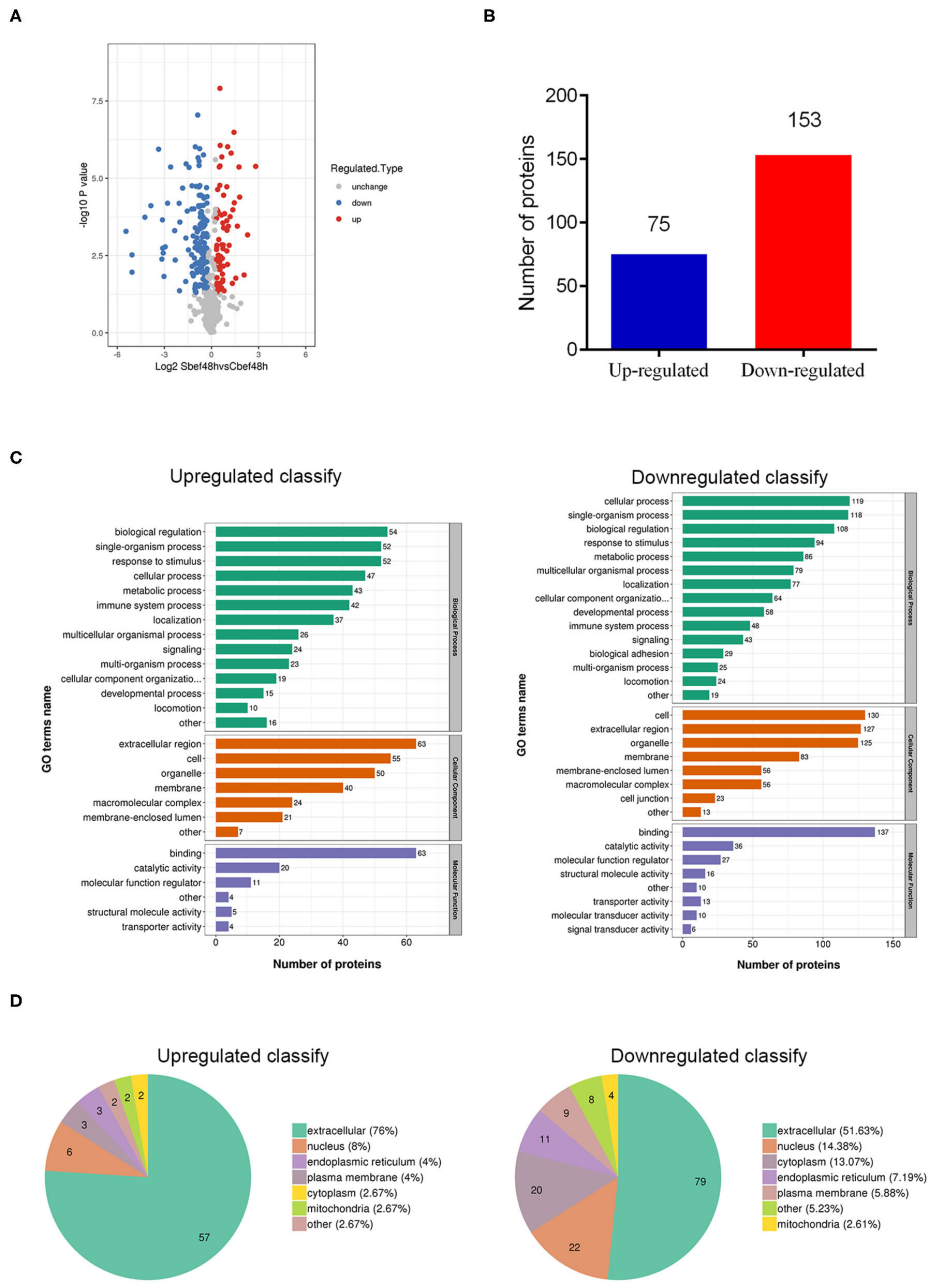
Overview of differentially expressed proteins in human plasma delivered exosomes. **(A)** The hot map of differently expressed proteins in exosomes. **(B)** Number of up-regulated and down-regulated proteins. **(C)** Gene Ontology functional analysis of differently expressed proteins in exosomes (biological process, cellular component, and molecular function). **(D)** Subcellular localization analysis of differently expressed proteins in exosomes.

Through quantitative proteomics analysis (detailed in Supplementary Material), we detected 590 quantifiable proteins, and 75 proteins were upregulated and 153 proteins were downregulated in human plasma exosomes (Sbef48h vs. Cbef48h, filtered with a threshold value of expression fold change>1.2 and *P* < 0.05) (Figures 2A,B). These differentially expressed proteins were investigated by Gene Ontology functional analysis to discover the biological process, cellular component, and molecular function (Figure 2C). Gene Ontology functional analysis showed that enriched biological process terms were related to biological regulation, single-organism process, cellular process, response to stimulus, and metabolic process. Gene Ontology functional analysis showed that enriched cellular component terms were in the extracellular region, cell, organelle, and membrane. Gene Ontology functional analysis showed that enriched molecular function terms were mainly related to binding, catalytic activity, and molecular function regulation. The subcellular localization analysis showed that these differentially expressed proteins were mainly in the extracellular region (Figure 2D). The statistical results of these data were consistent with the biological characteristics of human plasma exosomes.

### Functional Enrichment Analysis of Differentially Expressed Proteins in Human Plasma Delivered Exosomes

**Figure 3:**
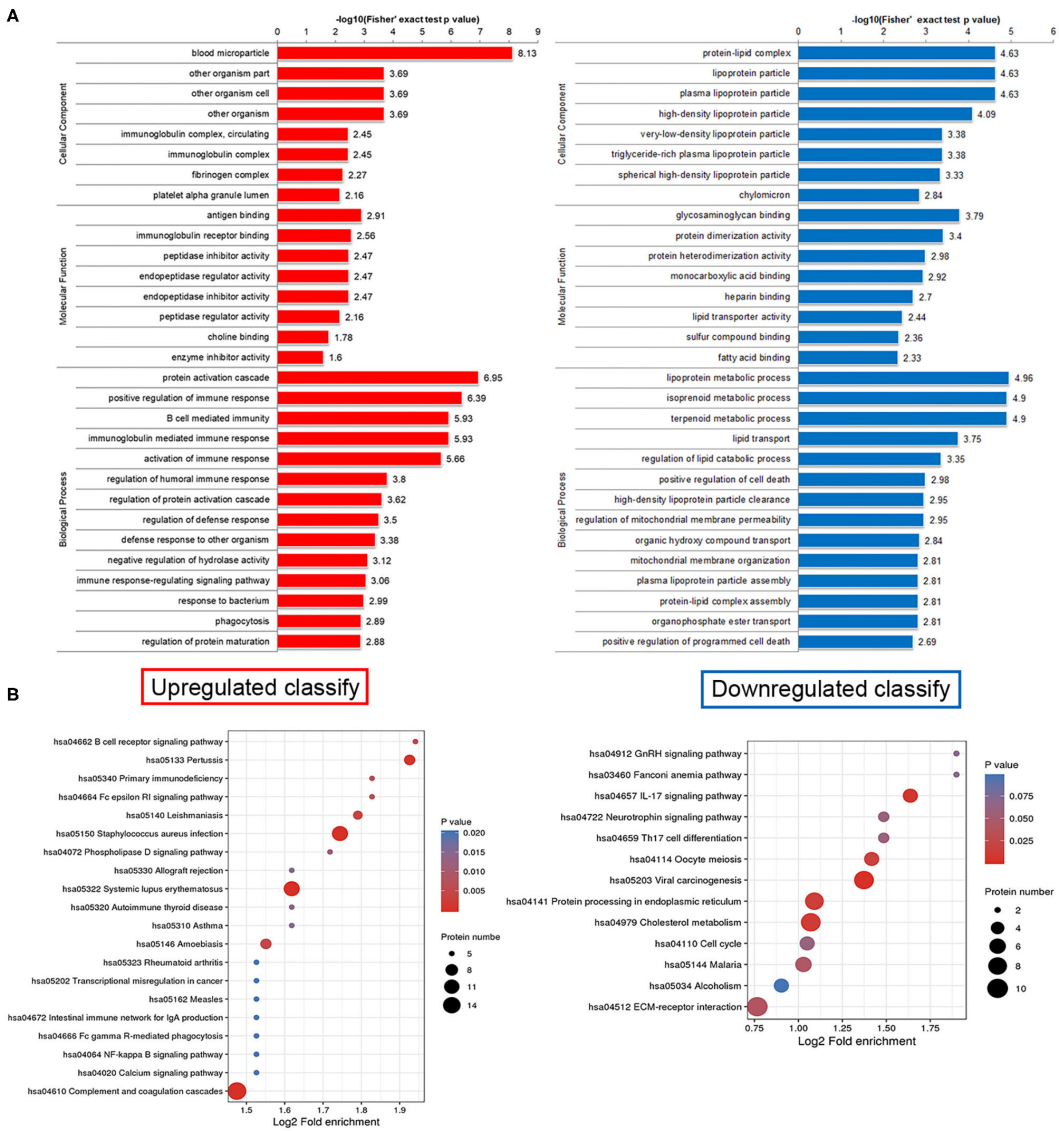
Functional enrichment analysis of differentially expressed proteins in human plasma delivered exosomes. **(A)** Enrichment analysis on both GO classification and KEGG pathway in each comparison group. **(B)** The hot map of KEGG pathway enrichment analysis. Left side, Upregulated clarify; Right side, Downregulated clarify.

This study lists the top 10 differentially expressed proteins in Table 2, including name, molecular weight, localization, and KEGG pathway number. To discover whether the differentially expressed proteins have a significant enrichment trend, we performed the enrichment analysis on both GO classification and KEGG pathway in each comparison group (Figure 3A). Surprisingly, cellular component enrichment analysis showed that all the downregulated proteins were related to lipoprotein particles. Molecular function enrichment analysis showed that parts of the downregulated proteins were related to lipid transporter activity. Biological process enrichment analysis showed that the lipoprotein metabolic process was on the top among the process of downregulated proteins. KEGG pathway enrichment analysis showed that the largest part of proteins was related to cholesterol metabolism among the downregulated proteins (Figure 3B).

**Table 2:** Top10 differentially expressed proteins and details.

**Protein accession**	**Gene name**	**Ratio**	**Regulated type**	**MW [kDa]**	**Subcellular localization**	**KEGG KO No**.
P62805	HIST1H4A	0.023	Down	11.367	Nucleus	K11254
Q71DI3	HIST2H3A	0.03	Down	15.388	Nucleus	K11253
Q16778	HIST2H2BE	0.03	Down	13.92	Nucleus	K11252
P14317	HCLS1	0.053	Down	54.013	Cytoplasm	K06106
P06702	S100A9	0.097	Down	13.242	Cytoplasm	K21128
Q99879	HIST1H2BM	0.112	Down	13.989	Nucleus	K11252
P05109	S100A8	0.119	Down	10.834	Mitochondria	K21127
P0DP25	CALM3	0.197	Down	16.837	Cytoplasm, nucleus	K02183
Q04917	YWHAH	0.202	Down	28.218	Cytoplasm	K16198
P31947	SFN	0.24	Down	27.774	Cytoplasm, nucleus	K06644
P05546	SERPIND1	1.783	Up	57.07	Extracellular	K03912
P02746	C1QB	1.986	Up	26.721	Extracellular	K03987
Q9NUV7	SPTLC3	2.029	Up	62.049	Nucleus	K00654
A0A0C4DH34	IGHV4-28	2.04	Up	13.124	Extracellular	K06856
P0C0L4	C4A	2.1	Up	192.78	Extracellular	K03989
P0DP08	IGHV4-38-2	2.177	Up	13.016	Extracellular	K06856
A0A087WSY4	IGHV4-30-2	2.736	Up	13.025	Extracellular	K06856
P46059	SLC15A1	3.133	Up	78.805	Plasma membrane	K14206
P0C0L5	C4B	3.464	Up	192.75	Extracellular	K03989
P63267	ACTG2	4.221	Up	41.876	Cytoskeleton	K12315

### Exosomes Involved in the Regulation of Cholesterol and Lipoprotein Metabolism After SCI in the Acute Phase

**Figure 4:**
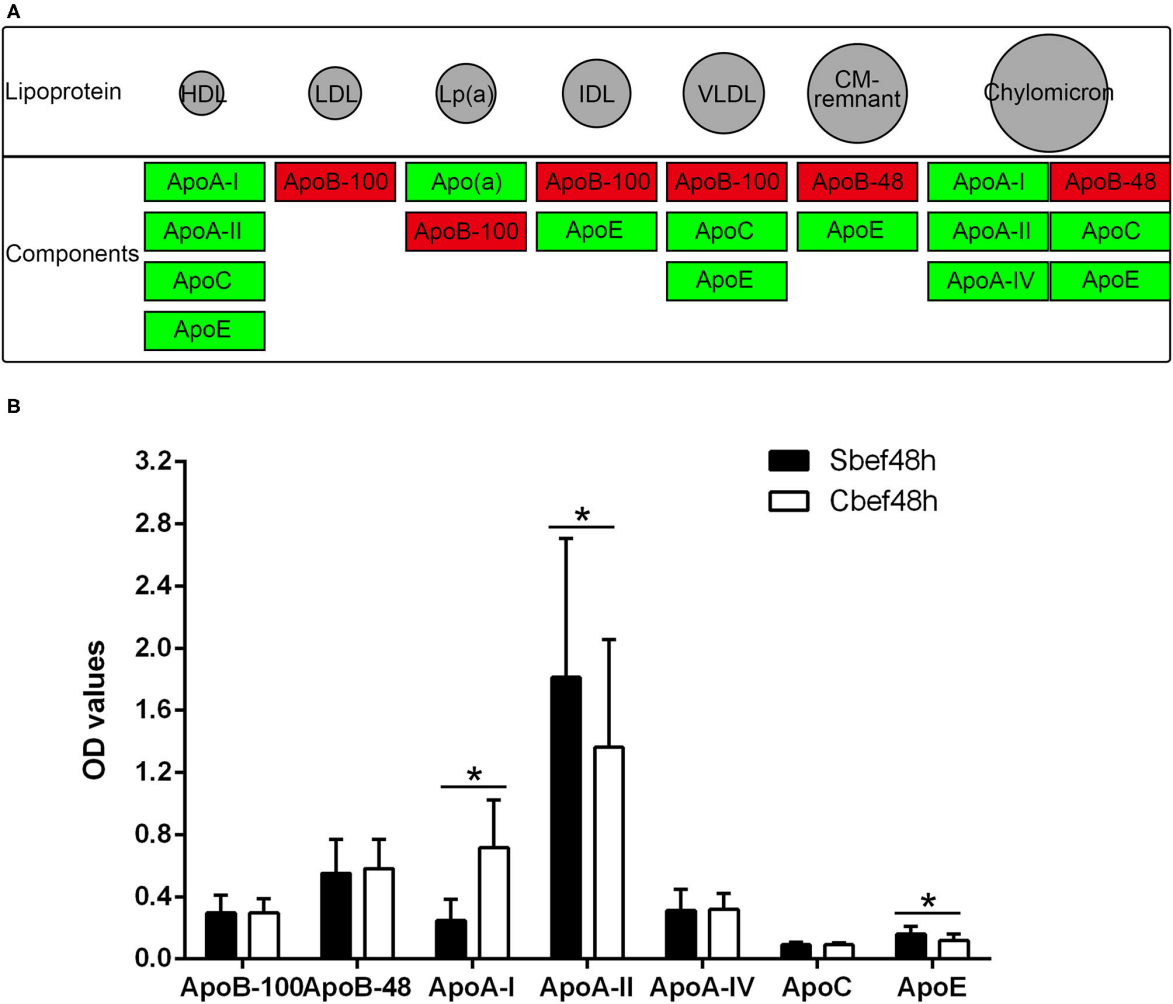
The cholesterol metabolism pathway and Elisa analysis of the components of cholesterol and lipoprotein. **(A)** hsa04979 KEGG pathway about the cholesterol metabolism (red, increased; green, decreased). **(B)** Elisa analysis of individual components of cholesterol in plasma. *P < 0.05.

The cholesterol metabolism pathway (hsa04979) is a signal pathway obtained by KEGG pathway enrichment analysis, which is closely related to downregulated proteins in exosomes. The expression of ApoB-48 and ApoB-100 increased, and the expression of ApoA-I, ApoA-II, ApoA-IV, ApoC, ApoE, and Apo(a) decreased in exosomes (Figure 4A). ELISA analysis is used to detect changes in individual components of cholesterol in plasma. Data showed that the concentration of some components was disordered in the acute phase of SCI (ApoA-I decreased, ApoA-II and ApoE even upregulated, ApoB-48, ApoB-100, ApoA-IV, and ApoC not changed) (Figure 4B). These results suggested that the expression of cholesterol and lipoprotein components had changed, and exosomes might be involved in their metabolism regulation in the acute phase of SCI.

## Discussion

Spinal cord injury is a common and severe disease caused by accidents and it would bring catastrophic consequences and huge economic burden to the patients. The treatment of SCI mainly relies on surgical operations and postoperative rehabilitation. However, the recovery of nerve function and the occurrence of complications are still unpredictable (Kreinest et al., 2016). During treatment, multiple system complications could occur, including respiratory failure, pneumonia, bedsores, urinary tract infection (UTI), thromboembolic diseases, and cardiovascular accidents (Maharaj et al., 2016). The incidence of coronary atherosclerotic heart disease (CHD) caused by abnormal lipid metabolism after SCI is as high as 12%, which is significantly higher than that of the general population (6%) (Aidinoff et al., 2017). Current serum lipid analysis shows that TC, LDL-c, VLDL-p, LDL-c, and TG are elevated, while HDL-c is reduced in patients with SCI (Koyuncu et al., 2017; La Fountaine et al., 2017). These studies are focused on the chronic phase of SCI, and it is believed that abnormal lipid metabolism is caused by long-term bed rest after paralysis. The main obstacle to the lack of studies on lipid metabolism in the acute phase of SCI is the BSCB (Sharma, 2005). Exosomes have relatively less limitation of BSCB within 48 h after SCI due to their smaller particle size, which makes them easier to be secreted to PB (Lu et al., 2019). Exosomes were used as research samples to analyze the lipid components in the PB of patients with SCI in the acute phase.

In this study, cholesterol and lipoprotein analysis of PB from patients with SCI in the acute phase showed that the clinically common indicators [ApoA, ApoB, Lp(a), TG, TC, HDL-c, and LDL-c] did not change significantly, which is different from the previous research results in chronic phase. Is this because of the relatively short bedtime or other reasons? We extracted exosomes from peripheral plasma from patients with SCI within 48 h and performed proteomics and bioinformatics analysis. Surprisingly, we found that all the downregulated proteins were related to lipoprotein particles and parts of them were related to lipid transporter activity. The expression of ApoB-48 and ApoB-100 increased, and the expression of ApoA-I, ApoA-II, ApoA-IV, ApoC, ApoE, and Apo(a) decreased in exosomes. RT-PCR analysis confirmed the results that the expression of these main components of lipid and cholesterol was changed in exosomes derived from the plasma of patients with SCI in the acute phase. These results suggested that exosomes in peripheral plasma had been already participated in regulating lipid metabolism in the acute phase of SCI. However, these results did not cause changes in the PB lipid concentrations. ELISA analysis of individual components of lipid showed that some components were disordered in the acute phase of SCI. This might be because the lipid components change caused by exosomes had not yet reached the corresponding threshold. The different expression trend of lipid components between exosomes with PB could be interpreted as a stress response that might be made in advance by exosomes for resisting external stimuli.

In addition, we also found the top 10 differentially expressed proteins in exosome derived from plasma of patients with SCI in acute phase through proteomics and bioinformatics analysis, including downregulating proteins: HIST1H4A, HIST2H3A, HIST2H2BE, HCLS1, S100A9, HIST1H2BM, S100A8, CALM3, YWHAH, and SFN, and upregulating proteins: SERPIND1, C1QB, SPTLC3, IGHV4-28, C4A, IGHV4-38-2, IGHV4-30-2, SLC15A1, C4B, and ACTG2. These proteins may also be involved in regulating lipid metabolism or other signaling pathways in the acute phase of SCI. Data results of this study suggested that the expression of cholesterol and lipoprotein components had changed, and exosomes might be involved in their metabolism regulation in the acute phase of SCI. These differentially expressed proteins might have been regulated at the gene level. Therefore, there must be a large number of differentially expressed genes in exosomes, which would be the next research content, including RNA-seq and biological informatics analysis.

## Data Availability Statement

The datasets presented in this study can be found in online repositories. The names of the repository/repositories and accession number(s) can be found in the article/Supplementary Material.

## Ethics Statement

The studies involving human participants were reviewed and approved by Ethics Committee of the Nantong First People's Hospital. The patients/participants provided their written informed consent to participate in this study.

## Author Contributions

All the authors confirmed they have contributed to the content of this paper and have completed the following three aspects: significant contributions to the experimental design and conception, acquisition, analysis, and interpretation of data, drafting or revising the article, and final approval of the published article.

## Conflict of Interest

The authors declare that the research was conducted in the absence of any commercial or financial relationships that could be construed as a potential conflict of interest.

## Abbreviations

SCI: Spinal cord injury
CNS: Central Nervous System
BSCB: Blood spinal cord barrier
BBB: Blood–brain barrier
TC: Total cholesterol
LDL-c: Low-density lipoprotein cholesterol
VLDL-p: Very low-density lipoprotein
TG: Triglyceride
HDL-c: High-density lipoprotein cholesterol
CM: Chylomicrons
CSF: Cerebrospinal fluid
PB: Peripheral blood.
